# Abnormal physiological responses toward sensory stimulus are related to the attention deficits in children with sluggish cognitive tempo

**DOI:** 10.3389/fnins.2022.875064

**Published:** 2022-08-23

**Authors:** Trevor W. K. Yung, Cynthia Y. Y. Lai, Chetwyn C. H. Chan

**Affiliations:** ^1^Department of Rehabilitation Sciences, The Hong Kong Polytechnic University, Kowloon, Hong Kong SAR, China; ^2^Department of Psychology, The Education University of Hong Kong, Hong Kong, Hong Kong SAR, China

**Keywords:** sensory modulation, sluggish cognitive tempo (SCT), attention, arousal regulation, negative valence, anxiety

## Abstract

Previous studies have found that sluggish cognitive tempo (SCT) is often associated with difficulties in real-life functioning, such as social problems, emotional difficulties, and academic learning difficulties. However, the underlying mechanisms contributing to the SCT symptoms and its associated real-life difficulties have still not been clearly understood. A previous study has found that SCT symptoms were associated with hypoarousal and hyperarousal toward the sensory stimulus. However, it is still unclear whether such abnormal arousal regulation is related to sustained attention difficulties that have been found to be related to social difficulties and withdrawn behavior in children with SCT. In this study, arousal regulation deficit in SCT is examined by the physiological responses quantified by HRV and EEG in the sensory challenge paradigm. This study aimed to establish a linkage between arousal regulation reflected by HRV and EEG and attention difficulties in children with SCT. The results of this study showed that higher theta power in the auditory stimulation condition than in the resting condition was associated with higher omission errors in sustained attention tasks in the SCT group. It was also found that higher parasympathetic activities during sensory stimulation conditions were associated with higher commission errors in the SCT group. These results reflected that hypersensitivity toward stressful sensitivity toward a stressful sensory stimulus is associated with attention difficulties in children with SCT. This further supported the notion that SCT should be conceptualized as a condition characterized by multiple deficits in different biological systems, such as the cognitive system, the negative valence system, and the arousal regulatory system.

## Introduction

“Sluggish cognitive tempo” (SCT) is a clinical condition that was found to be characterized by excessive daydreaming and sleepiness as well as slowness in thoughts and actions (Penny et al., 2009; Barkley, 2014; Becker et al., 2016a). These behavioral symptoms are often associated with real-life impairments, such as emotional difficulties (e.g., anxiety and depression) (Smith and Langberg, 2017; Becker et al., 2018a; Servera et al., 2018; Burns and Becker, 2019), social problems (Becker, 2014; Becker et al., 2014; 2018a), and academic learning difficulties (Langberg et al., 2014; Tamm et al., 2016). Growing findings have shown that SCT is distinct from any form of attention deficit/hyperactivity disorder (ADHD) (Garner et al., 2014; Willcutt et al., 2014; Becker et al., 2016b; Lee et al., 2016). However, the underlying mechanism contributing to the symptoms, the emotional, and social difficulties in individuals with SCT has still not been clearly understood.

### Abnormality in sensory modulation abilities reflect underlying mechanism causing sluggish cognitive tempo symptoms

Sensory modulation can be defined as the ability to adjust one’s physiological or behavioral responses toward sensory stimulation. Sensory over-responsivity and under-responsivity are two patterns of sensory modulation difficulties and are often associated with over-arousal and under-arousal, respectively. Given the “drowsy,” “slow,” and “daydreamy” nature of SCT behavioral symptoms, previous researchers have suggested that SCT may be related to difficulties in arousal/regulatory processes (Barkley, 2014; Becker and Willcutt, 2018). Those commonly seen difficulties in SCT, such as inattention, anxiety/depression, and withdrawn behavior, have been suggested to be related to hypo- and/or hyperarousal (Becker and Willcutt, 2018). In fact, these hypo- and hyperarousal states can be reflected by autonomic nervous system (ANS) activity. Previous studies have found that there was reciprocal action between the locus coeruleus (LC) and ANS (Samuels and Szabadi, 2008; Wood and Valentino, 2017). Therefore, activity in the LC could induce changes in cardiovascular responses (Wang et al., 2014). All these evidences supported a reciprocal pathway between the LC and ANS, and thus we can understand the arousal/regulatory processes in individuals in SCT by investigating the ANS activities among them. In fact, previous studies have found that the ANS plays a very crucial role in the regulation of physiological arousal during executive functioning and attention tasks (Suess et al., 1994; Thayer and Lane, 2000; Lesley et al., 2014; Zahn et al., 2016). Therefore, it is possible that abnormal ANS activities may reflect deficient arousal/regulatory processes in individuals with SCT, and subsequently lead to attention and executive functioning difficulties, which subsequently cause difficulties in daily functioning. However, no previous study has examined such relationships between arousal/regulation and SCT symptoms; only one study has examined the neurophysiological correlates of SCT symptoms using different ANS measurements (Yung et al., 2020).

The study by Yung et al. (2020) was the first to examine the neurophysiological correlates of SCT symptoms among children using ANS measures. In their study, they conceptualized that abnormalities in ANS readiness and ANS regulation would reflect the deficient arousal state and regulatory processes in individuals with SCT (Yung et al., 2020). ANS readiness was defined as the physiological readiness in ANS of an individual to prepare for the challenges in the real life, while ANS regulation was defined as the ability to regulate one’s physiological activity when an individual is dealing with real-life challenges. Yung et al. (2020) recruited 30 children aged 6–12 years and measured their heart rate variability (HRV) during resting and warning signal conditions. HRV measures in the resting condition would reflect one’s physiological readiness state, whereas HRV measures in the warning signal condition would reflect one’s physiological regulation when facing challenges. Yung et al. (2020) found that SCT symptoms were positively and significantly associated with resting SD2 nu (the standard deviation of the Poincare plot along the line of identity in normalized units of HRV) and with changes in SD2 nu and pNN50 (the percentage of successive RR intervals that differ by more than 50 ms) between the resting and warning signal conditions. The results of Yung et al.’s (2020) study (2020) suggested that SCT symptoms were related to the ANS readiness and ANS regulation, which reflected the arousal state and regulatory processes, respectively, in children with SCT. It may also reflect that people with high-SCT symptoms may have heightened arousal in response to pure tone auditory stimulus (Yung et al., 2020).

### Cortical activities, autonomic nervous system functions, and attention deficits in sluggish cognitive tempo

Based on the neurovisceral integration model (Thayer and Lane, 2000; Thayer et al., 2009), cognitive functions and autonomic functions are interrelated processes. The central autonomic network (CAN) has been suggested as a crucial component of an internal regulation system in the ANS through which the brain controls viscera-motor, neuroendocrine, and behavioral responses. All these responses support goal-directed behavior (Thayer et al., 2009). This model also suggested a biological bidirectional connection between the prefrontal cortex (PFC) and ANS. PFC could exert influence on subcortical structures (e.g., amygdala and thalamus) so that an individual could control the psychophysiological resources in attention and executive functions to organize his/her behavior and deal with the challenge in the environment (Thayer et al., 2009). Therefore, PFC functions and ANS functions are two interrelated processes that support our attention to deal with real-life challenges. However, no previous study has examined the linkage between cortical activities, ANS functions, and attention difficulties in children with high-SCT symptoms. Therefore, it is unclear whether the abnormalities in ANS functions and cortical activities during sensory challenge situation could explain the attention difficulties among these children. The aim of this study was to examine the relationship between arousal regulation (reflected by cortical and ANS activities) and attention difficulties among children with high-SCT symptoms. As there is no previous research related to the relationship between arousal regulation and attention difficulties in children with high-SCT symptoms, no prior hypotheses could be made in this study. Given there is a strong link between arousal and sustained attention, it is expected that both abnormalities in cortical activities and ANS activities during auditory stimulation conditions would be significantly associated with attention difficulties among children with SCT.

## Materials and methods

### Participants

The participants involved in this study were required to meet the following inclusion criteria: (a) aged 6–13 years and (b) a full-scale IQ score > 80. Potential participants who have been given a psychiatric diagnosis by psychiatrists and/or psychologist, including autism spectrum disorder, ADHD, oppositional defiant disorder, and conduct disorder were excluded as these disorders have been found to be associated with different neurophysiological and neuropsychological deficits. In other words, these conditions could have confounded the relationship between the neurophysiological and social problems.

Eighty-eight primary school students aged 6–13 years in Hong Kong were recruited *via* posters sent to primary schools and parent chat groups. Among them, 41 children [mean age = 111.22 months, standard deviation (*SD*) = 19.032; 47.1% female] were classified as the SCT group using the median split method on the score of the SCT scale (Penny et al., 2009). Forty-seven children [mean age = 109.23 months, standard deviation (*SD*) = 18.042; 46.8% female] were classified as the Control group.

### Procedure and experimental setup

All participants were recruited from primary schools and parent chat groups. A research package (information on the study and consent form) was given to the parents of the participants. Written informed consents of all participants were obtained before the data collection. The researcher contacted the parents of all participants to collect their basic demographic information (e.g., name, date of birth, and gender) and the medical history of their children. All participants were invited to attend a 2–3 h testing session (breaks were provided between assessments) at the university laboratory to receive the neuropsychological tests and neurophysiological measures (to be described in detail in the “Measuring Instruments” section). Parents of the participants were required to fill in the rating scales (to be described in detail in the “Measuring Instruments” section) in the waiting room when their children were receiving testing in the laboratory.

Participants received the neuropsychological testing in a room without any visual or auditory distractions. Short breaks were offered to the participants between tests. As HRV measurement would be easily affected by the condition of the testing room, such as illumination and temperature, the condition of the experiment laboratory room was set as follows during all the neurophysiological measurements: (a) illumination level was set to 10 lux; (b) room temperature was set to 23–25°C; and (c) background noise level at 40–45 dB. Before the HRV measurements, all participants were reminded not to do the following: (a) intake caffeinated drinks before the testing; (b) engaged in any rigorous activities 24 h before the testing; and (c) receive any treatment or medication that could affect ANS activity; as all these events may affect the accuracy of the HRV measurement. It is suggested that HRV measurement can be affected by the circadian rhythm. Previous studies have found that a decrease in HRV during the course of a day is closely associated with the increased SNS activity during the evening and first few hours of the night (D’Negri et al., 2005; Sinha et al., 2021). The HRV parameters between 10 am and 5 pm are relatively stable in these two studies. In this study, HRV measurements were conducted between 10 am and 5 pm, thus the results of this study would not be influenced by the circadian rhythm.

### Measuring instruments

In this study, neuropsychological tests and neurophysiological measures were applied to children who participated in the research. All participants were invited to finish all the neuropsychological measures first and then to finish the neurophysiological measures. Sufficient breaks were offered to each participant before the neurophysiological measures to minimize the possible fatigue effect caused by the administration of neuropsychological measures.

### Neuropsychological tests

• *SWAN—Chinese version (Parent Version).* SWAN is a parent rating scale used to screen for ADHD symptoms (Swanson et al., 2012.) The Chinese-translated version used in this study has been validated for use with children in Hong Kong (Education Bureau of HKSAR, 2010). The scale comprises 18 items that measure a child’s control of their attention, impulses, and activity. The items are divided into two subscales of nine items each, which address inattention and hyperactivity/impulsivity, respectively. The total and subscale scores are generated by summing the raw item scores, which can be expressed as T-scores. A higher score indicates fewer ADHD symptoms. In this study, only the total SWAN scores were used. All of the total and subscale scores for both the parent and teacher versions of SWAN have been found to yield a very good internal consistency (alpha > 0.9) and a good discriminant validity (AUC > 0.8) (Education Bureau of HKSAR, 2010).
• *SCT Scale* (Penny et al., 2009). This 14-item parent scale is used to measure SCT symptoms in children. Each item is rated on a 7-point scale (range: 0 = *not at all* to 6 = *very much*). Three subscale scores were obtained from the SCT scale: SCT Daydream subscale score, SCT Sleepy subscale score, and SCT Slow subscale score. SCT Daydream subscale included items, such as *“get lost in his or her own thoughts”* and *“seems to be in world of his own,”* while the SCT Sleepy subscale included items, such as *“seems drowsy”* and *“appears tired; lethargic.”* SCT Slow subscale included items, such as *“lacks initiate to complete work”* and *“is apathetic; shows little interest in things or activities.”*

### Neurophysiological measures

• *ANS measures.* HRV is a promising method to quantify ANS activity (Task Force of the European Society of Cardiology, 1996). In this study, HRV was measured to examine the SNS and PNS activities across different experimental conditions (resting condition and warning signal condition; refer to the “Experimental Paradigm” section for details). Polar H2 Heart Rate Monitors were considered as valid tools to measure HRV (de Rooij et al., 2013).
• *EEG measures.* EEG frequency measures were used to quantify cortical activities during the experimental condition and warning signal condition. During the experiment, electrodes and transducers were applied to the participants and connected to a BIOPAC MP360 system to record EEG signals. EEG was recorded from the electrode at Cz and a linked earlobe reference was used. EEG electrodes were connected to the Biopac EEG amplifier, which was set to bandpass filter between 0.1 and 100 Hz and a sampling frequency of 1,000 Hz.

### Experimental paradigm

#### Warning signal paradigm

The experimental paradigm of this study was the same as that of Yung et al.’s (2020). There were two conditions in this experimental protocol: resting condition and warning signal condition. HRV and EEG were measured continuously across these two conditions. During the resting condition, a silent cartoon movie was shown to the participants for 200 s. After that, the monitor screen turns blank and the participants received a block of warning signal conditions (a block of 10 trials of a 4 kHz pure tone at 85 dB and each trial lasting for 3 s). To avoid adaptation to the stimulus, a pseudorandomized interval was set from 10 to 15 s.

### Cued continued performance test

The cued continued performance test (CCPT) was used to measure the performance of sustained attention ability in this study. The CCPT paradigm is made reference to that of Nash et al. (2013). The subjects were required to press the response button when the target stimuli appeared on the computer screen, while required to withhold from responding when the non-target stimuli appeared. Stimuli were letters presented at the center of the computer screen (one letter at a time for 200 ms with an ISI of 1,650 ms) in a pseudo-randomized order. Target stimuli were a paired sequence of stimuli in which the letter O was presented first, followed by the letter X. In the sequence of non-target stimuli, the letter O was presented first, followed by non-X letters. The stimulus set consisted of two blocks of 200 trials, and the subjects were asked to take a 1-min break after the first block to avoid mental fatigue. In each block, 40 stimuli were “O letter” stimuli, 20 were target stimuli, and 20 were non-target stimuli. The remaining 120 stimuli were distractor letters (letters other than O or an X without a preceding O). The measures generated from each block of the tests were the numbers of target hits (X with a preceding O), omission errors (no response toward the target), commission errors (response to non-target and distractor stimuli), and response latency toward the target. The final measures used in the analysis in this study were calculated by deducting the value of each measure in block 1 from the value of that measure in block 2 (i.e., the final measure of omission errors = the number of omission errors in block 2—the number of omission errors in block 1). The rationale behind such calculation was to obtain a measurement of performance deterioration over time, which was assumed to reflect sustained attention (Wåhlstedt and Bohlin, 2010).

### Signal processing

#### Heart rate variability signal

All the HRV raw data were converted into a tachogram by using aHRV (Nevrokard, Slovenia). Tachograms were visually scanned for ectopic beats, movement artifacts, and abnormal noise signals. Data were then epoched into specific time events in the experimental paradigm of this study. Values of 20% under or over the mean of the preceding 25 beats will be treated as artifacts for the short-term recording. Identified artifacts were then edited by using interpolation. Data with more than 3% correction from the total normalized HRV data samples were discarded from the analysis, as recommended by the Task Force of the European Society of Cardiology (1996). HRV is defined as the variation in the time intervals between adjacent heartbeats (Shaffer and Ginsberg, 2017). In this study, non-linear measurement of HRV was used. Non-linear measurement of the HRV index quantifies the unpredictability of a time series, which results from the complexity of the sympathovagal mechanism that regulates the HRV (Shaffer and Ginsberg, 2017). In the non-linear measurement, SD1 and SD2 of Poincare Plot are commonly used. SD1 is the Poincare Plot standard deviation perpendicular to the line of identity and was suggested to reflect short-term variability of HRV reflecting PNS activity. SD2 is the Poincare plot standard deviation along the line of identity and is suggested to reflect the long-term variability of HRV (Tulppo et al., 1996; Brennan et al., 2001; Guzik et al., 2007; Shaffer and Ginsberg, 2017). Previous researches have shown that SD2 is negatively related to sympathetic influence on the heart (Sharma et al., 2009; Negrao et al., 2011; Goit and Ansari, 2016; Rahman et al., 2018).

#### EEG signal

The EEG raw signal was processed by the Biopac Acknowledge 5.0 Software. EEG raw signal at Cz was first filtered using IIR band pass filter (low cut off = 0.5 Hz and high cut off = 44 Hz) and was generated into the following standard EEG bands: Delta (0.5–4 Hz), theta (4–8 Hz), alpha (8–13 Hz), beta (13–30 Hz), and gamma (30–44 Hz). The power spectral density function was used to estimate the power spectrum of each 3-s epoch using a Welch periodogram estimation method. The mean power of all epochs was averaged and was used to represent the power of each EEG brand in two experimental conditions.

## Results

### Associations between EEG variables and the cued continued performance test measures

Bivariate correlation analysis was also conducted to examine the correlations between EEG variables [Resting EEG Theta power, Resting EEG Alpha power, EEG Theta power (Warning Minus Resting), and EEG Alpha power (Warning Minus Resting)] and CCPT measures (CCPT Omission, CCPT Commission, and CCPT Response Time). The mean and standard deviation of EEG measures and CCPT measures in the SCT and the Control group are shown in Table 1. In the SCT group, EEG Theta power (Warning minus Resting) and EEG Alpha power (Warning minus Resting) were both found to have a significant positive relationship with CCPT Omission (Table 2). In the control group, no significant correlation was found between the EEG measures and CCPT measures.

**TABLE 1:** Mean and standard deviation of EEG measures and CCPT measures in the SCT and control groups.

	SCT group	Control group
	*M* (*SD*)	*M* (*SD*)
Omission	1.13 (2.59)	0.91 (2.09)
Commission	2.15 (27.64)	1.46 (9.29)
Response time	34.46 (158.34)	54.19 (103.53)
Resting EEG Theta	0.000048 (0.000056)	0.000026 (0.000012)
Resting EEG Alpha EEG Theta (Warning minus resting) EEG Alpha (Warning minus resting)	0.000025 (0.000023) 0.000023 (0.0001) 0.0000097 (0.000041)	0.000015 (0.000007) 0.00000041 (0.0000057) 0.00000087 (0.0000032)

CCPT, Cued continued performance test; SCT, Sluggish cognitive tempo; *M*, Mean; *SD*, Standard deviation.

**TABLE 2:** Correlation coefficients of the CCPT measures with the EEG measures in the SCT group.

	CCPT measures		
	
	Omission	Commission	Response time
Resting EEG Theta	0.287	0.035	−0.139
Resting EEG Alpha EEG Theta (Warning minus resting) EEG Alpha (Warning minus resting)	0.197 0.495* 0.461*	0.023 −0.131 −0.112	−0.063 0.011 −0.003

CCPT, Cued continued performance test; CBCL, Child behavior checklist; SCT, Sluggish cognitive tempo; Correct, CCPT correct response, Omission, CCPT omission errors; Commission, CCPT commission errors; Response time, CCPT response time. *p < 0.05.

### The associations between EEG variables and the cued continued performance test omission after controlling attention deficit/hyperactivity disorder symptoms

A regression analysis was again performed to examine the associations between the EEG variables and the CCPT Omission after entering the SWAN ADHD score into the model to control for ADHD symptoms (Step 1). EEG Theta power (Warning minus Resting) was then entered into the model (Step 2). Finally, EEG Alpha power (Warning minus Resting) was entered into the model (Step 3) to explain the variance of the CCPT Omission. An *a priori* power analysis was conducted using the G*Power version 3.1 to determine the minimum sample size required to test the hypothesis in this study. Results indicated the required sample size to achieve 80% power for detecting a medium effect at a significance criterion of α = 0.05 was 36 for regression analysis in this study. Therefore, the obtained sample size is adequate to test the current hypothesis. In the SCT group, the SWAN ADHD score did not contribute significantly to the regression model to explain the CCPT Omission [*F*(1,20) = 0.703, *p* > 0.05] (Table 3). EEG Theta power (Warning minus Resting) was found to be the only significant factor to explain the variance of CCPT Omission [*F*(2,19) = 4.085, *p* < 0.05] (Table 3). This factor explained 30.1% of the variance in CCPT Omission (Table 3).

**TABLE 3:** Regression model of the EEG measures as the predictors of the CCPT omissions in the SCT group.

Variable	Unstandardized B	Coefficient standard error	Standardized coefficient beta	*t*	Sig.	*R*	*R* ^2^	Adjusted *R*^2^	*R*^2^ change
**Step 1**									
SWAN ADHD score	0.028	0.033	0.184	0.838	0.412	0.184	0.034	−0.014	0.034
**Step 2**									
SWAN ADHD score	0.017	0.029	0.109	0.565	0.579	0.548	0.301	0.227	0.267*
EEG Theta (Warning minus resting)	10,652	3,956	0.522	2.692	0.014				
**Step 3**									
SWAN ADHD score	0.018	0.029	0.117	0.615	0.546	0.601	0.361	0.254	0.060
EEG Theta (Warning minus resting)	−22,538	25,801	−1.1	−0.874	0.394				
EEG Alpha (Warning minus resting)	86,708	66,634	1.643	1.3	0.210				

CCPT, Cued continued performance test; SCT, Sluggish cognitive tempo; ADHD, Attention-deficit hyperactivity disorder; SWAN, the Strength and Weaknesses of ADHD Symptoms and Normal Behavior scale. *p < 0.05.

### The associations between heart rate variability variables and the cued continued performance test measures

Bivariate correlation analysis was conducted to examine the correlations between HRV variables (SD1 nu Resting, SD2 nu Resting, SD1 nu Warning minus Resting, and SD2 nu Warning minus Resting) and CCPT measures (Omission errors, Commission errors, and Reaction Latency) in both the SCT and control group. The means and standard deviations of the CCPT measures and the HRV variables of both groups are shown in Table 4. In the SCT group, CCPT Commission was found to be significantly and negatively associated with Resting SD1 nu and Resting SD2 nu (Table 5). It was also found that CCPT Commission was significantly and positively associated with SD1 nu (Warning minus Resting) (Table 5). In the Control group, no significant correlation was found between HRV variables and the CCPT measures.

**TABLE 4:** Mean and standard deviation of HRV measures and CCPT measures in the SCT and Control groups.

	SCT group	Control group
	*M* (*SD*)	*M* (*SD*)
Omission	1.13 (2.59)	0.91 (2.09)
Commission	2.15 (27.64)	1.46 (9.29)
Response time	34.46 (158.34)	54.19 (103.53)
Resting SD1 nu	6.12 (2.07)	5.81 (1.99)
Resting SD2 nu SD1 nu (Warning minus resting) SD2 nu (Warning minus resting)	10.77 (3.03) −0.25 (1.43) 0.87 (3.13)	9.33 (2.39) 0.22 (1.04) 1.76 (2.09)

CCPT, Cued continued performance test; SCT, Sluggish cognitive tempo; *M*, Mean; *SD*, Standard deviation.

**TABLE 5:** Correlation coefficients of the CCPT measures with the HRV in the SCT group.

	CCPT measures		
	
	Omission	Commission	Response time
Resting SD1 nu	−0.132	−0.429**	0.195
Resting SD2 nu SD1 nu (Warning minus resting) SD2 nu (Warning minus resting)	−0.046 0.202 −0.031	−0.340* 0.625** 0.083	0.277 −0.08 0.01

CCPT, Cued continued performance test; CBCL, Child behavior checklist; SCT, Sluggish cognitive tempo; Omission, CCPT omission errors; Commission, CCPT commission errors; Response time, CCPT response time. *p < 0.05, **p < 0.01.

### The associations between heart rate variability variables and the cued continued performance test commissions errors after controlling attention deficit/hyperactivity disorder symptoms

A regression analysis was performed to examine the associations between the CCPT Commission and the HRV variables after entering the SWAN ADHD score into the model to control for ADHD symptoms (Step 1). SD1 nu (Warning minus Resting) was then entered into the model (Step 2). Finally, SD1 nu and SD 2 nu were entered into the model stepwise (Step 3) to explain the variance of the CCPT Commission. An *a priori* power analysis was conducted using G*Power version 3.1 to determine the minimum sample size required to test the hypothesis in this study. Results indicated the required sample size to achieve 80% power for detecting a medium effect at a significance criterion of α = 0.05 was 41 for the regression analysis in this study. Therefore, the obtained sample size is adequate to test the current hypothesis.

In the SCT group, the SWAN ADHD score did not contribute significantly to the regression model to explain the CCPT Commission [*F*(1,37) = 0.101, *p* > 0.05] (Table 6). The regression model only identified the SD1 nu (Warning minus Resting) as a significant factor to explain the variance of the CCPT Commission (Table 6). This factor explained 39.4% of the variance in CCPT Commission [*F*(2, 36) = 11.72. *p* < 0.05].

**TABLE 6:** Regression model of the ANS measures as the predictors of the CCPT commissions in the SCT group.

Variable	Unstandardized B	Coefficient standard error	Standardized coefficient beta	*t*	Sig.	*R*	*R* ^2^	Adjusted *R*^2^	*R*^2^ change
**Step 1**									
SWAN ADHD score	0.10	0.315	0.052	0.318	0.75	0.052	0.003	−0.024	0.003
**Step 2**									
SWAN ADHD score	–0.118	0.253	–0.061	–0.466	0.644	0.628	0.394	0.361	0.392**
SD1 nu (Warning minus resting)	12.32	2.554	0.636	4.824	0.000				
**Step 3**									
SWAN ADHD score	−0.195	0.249	−0.102	−0.783	0.439	0.668	0.446	0.398	0.080
SD1 nu (Warning minus resting)	11.503	2.519	0.594	4.567	0.000				
Resting SD2 nu	−0.215	1.191	−0.236	−1.8	0.080				

CCPT, Cued continued performance test; SCT, Sluggish cognitive tempo; ADHD, Attention-deficit hyperactivity disorder; SWAN, the Strength and Weaknesses of ADHD Symptoms and Normal Behavior scale; Commission, CCPT Commission errors. **p < 0.01.

## Discussion

This study aimed to examine the relationship between abnormalities in arousal regulation reflected by EEG and HRV and attention difficulties in children with high-SCT symptoms. As expected, attention difficulties among these children were found to be significantly associated with EEG/ANS abnormalities during the sensory challenge conditions. Specifically, Theta power (Warning minus Resting) was found to be significantly associated with CCPT Omission after controlling ADHD symptoms. SD1 nu (Warning minus Resting) was also found to be significantly associated with CCPT Commission after controlling ADHD symptoms. CCPT Omission and CCPT Commission typically reflected deficits in sustained attention and inhibition, respectively. These two neuropsychological processes have been found to be significantly associated with SCT symptoms after controlling ADHD symptoms (Wåhlstedt and Bohlin, 2010; Willcutt et al., 2014; Baytunca et al., 2018; Creque and Willcutt, 2021). No previous study has examined what neurophysiological factors may contribute to their deficits in these two processes. The results of this study may fill this gap in this research on SCT.

### The association between theta power during warning signal task and cued continued performance test omission errors in sluggish cognitive tempo

In this study, a higher magnitude of theta power during the warning signal condition was found to be associated with higher omission errors in CCPT. Theta wave has been found to be generated by the anterior cingulate cortex (ACC) and was often associated with the neural processes of conflict monitoring and resolution (Cavanagh et al., 2009; Nigbur et al., 2011; Eschmann et al., 2018). For example, Cavanagh et al. (2012) have found that mid-frontal theta power increased in the conditions of novelty, conflict, punishment, and error. Active avoidance of potentially aversive outcomes has been found to be associated with theta signals that originated from the cingulate cortex (Cavanagh and Shackman, 2015). In this study, participants were presented a series of unexpected and high-pitched monotone during the warning signal condition. This research paradigm may create a situation for the participants in which there were uncertainty (i.e., unexpected auditory stimulus) and goal conflicts (wanted to escape from the aversive stimulus vs. had to remain on their seat during the experiment). The challenge posed by this situation may activate the anterior cingulate cortex and subsequently enhance the power of the theta wave.

According to Gray and McNaughton’s (2000) reinforcement sensitivity theory (2000), Behavioral Inhibition System (BIS) is a brain system that activates responses of inhibition and avoidance when aversive consequences are expected. Theta power has been found to be a significant neurophysiological factor in discriminating individuals with high/low sensitivity to Behavioral Inhibition System (BIS) (Moore et al., 2012). Individuals with higher sensitivity to BIS tended to have higher theta power during the goal-conflict tasks (Moore et al., 2012). BIS has been found to be significantly and positively associated with SCT symptoms using a behavioral rating scale (Becker et al., 2013, 2018b). No previous study has been conducted to provide neurophysiological evidence to support this association. Therefore, this study was the first evidence at the neurophysiological level which support the linkage between hypersensitivity to BIS and SCT symptomology. Specifically, the results of this study suggested that omission error, the common sustained attention difficulty in SCT, was related to the hypersensitivity to the BIS. High anxiety has been found to be associated with high sensitivity to BIS. Therefore, it is possible that a higher level of state anxiety, which is often associated with BIS hypersensitivity, may cause difficulty in attention. According to the Attention Control Theory (ACT), anxiety may increase the influence of stimulus-driven attentional systems and decrease the influence of goal-directed attention system in an individual (Eysenck et al., 2007). As the goal-directed attention system fails to override the stimulus-driven attentional systems, individuals tend to direct their attentional resources toward salient and conspicuous stimuli to detect threat-related information (Eysenck et al., 2007). As a result, less attentional resources would be directed to process goal-related information or stimulus and task performance would be negatively affected. In this study, individuals with high SCT, possibly due to their high sensitivity to BIS, may experience more anxiety that directs their attention toward the salient non-target (e.g., any letters) stimulus during the task. Subsequently, they failed to direct attentional resources toward inconspicuous target stimulus (e.g., X and then O) and committed more omission errors.

The results of this study also bring new evidence to support the argument that individuals with sluggish cognitive tempo are not really “sluggish” in general. Some studies have found individuals with SCT had significantly slower processing speed than typical individuals (Willcutt et al., 2014; Tamm et al., 2018), while other studies did not find significant results (Skirbekk et al., 2011; Bauermeister et al., 2012). Kofler et al. (2019) did not find significant associations between cognitively modeled information processing speed (drift rate) and SCT symptoms. This suggested that SCT symptoms were not related to general processing speed. However, they found that faster inhibition speed was significantly related to parent-reported SCT symptoms, and they interpreted this result as evidence to support that children with SCT are behaviorally over-inhibited and SCT symptoms were related to increased BIS sensitivity (Kofler et al., 2019). The results of the current study provided further neurophysiological evidence to support this notion. Our results have indicated that increased BIS sensitivity (higher theta wave during sensory challenge situation) would negatively affect children with SCT to allocate their attentional resources toward non-salient but relevant information in the task. Therefore, it is likely that increased BIS sensitivity may affect these children’s inhibition abilities in the similar way. For example, anxiety may increase the influence of the stimulus-driven attentional system, which drives these individuals more attentive toward the salient signal (the auditory signal indicating to stop) in the stop signal task, which subsequently enhances their inhibition speed. With such a tendency, individuals with SCT tend to be over-sensitive toward salient but irrelevant environmental information, while tend to be slow to respond to inconspicuous but relevant information in real life. In summary, children with SCT did not seem to respond slowly to all information in the real life. They tend to respond slowly to the target information if this information is threat-related and/or there are competing distractions that are threat-related in nature.

Omission errors in sustained attention task have been found to be positively associated with social problems and withdrawn behavior that are commonly present in children with high-SCT symptoms (Yung et al., 2021). The results of the current study provide new evidence to explain the linkage between sustained attention difficulty and social problems/withdrawn behavior. The current results supported the notion that omission errors were related to higher sensitivity to BIS in children with SCT. They tend to allocate their attentional resources to look for salient and potentially threatening information in the social environment. Subsequently, they assign relatively fewer resources to crucial but non-salient cues embedded in social interaction and thus make less appropriate social responses in a social situation. The results of this study provide a new perspective into the nature of the social difficulties experienced by children with high-SCT symptoms.

### The association between heart rate variability and cued continued performance test commission errors in sluggish cognitive tempo

This study has found that SD1 nu (Warning minus Resting) was found to be significantly associated with CCPT Commission. SD1 nu has been suggested to reflect parasympathetic activity in ANS. During stressful events, activation of parasympathetic activity results in heart rate deceleration, which was a core feature of freezing. Such stress-induced freezing may facilitate making an appropriate response by allowing further information gathering (Livermore et al., 2021). For example, it has been found that heart rate deceleration in heart rate during response preparation was related to the facilitation of perceptual decisions (Ribeiro and Castelo-Branco, 2019). Individuals with higher levels of state anxiety tended to have a higher freezing reaction toward angry faces (Roelofs et al., 2010) and SCT symptoms were often associated with higher anxiety level. Therefore, it is possible that higher CCPT commission errors may be associated with a higher propensity of freezing in children with high-SCT symptoms.

Why freezing during a stressful event is associated with higher commission errors of CCPT in children with SCT? The possible answer to this question may be that freezing is often associated with altered perceptual sensitivity. Heart rate deceleration was found to be associated with improved detection of low-spatial frequency (LSF) cues at the expense of high-spatial frequency (HSF) detection (Lojowska et al., 2015). Such an increase in the sensitivity toward LSF may promote individuals to detect coarse threat-relevant features of the object such as presence or location instead of the details of the visual presentation of the object (Bocanegra and Zeelenberg, 2009). It is possible that children with high SCT, due to their BIS sensitivity, tend to utilize stimulus-driven attention systems to detect salient threat-related information instead of the underlying goals. Therefore, they may be more prone to make responses whenever they saw “O” in the CCPT and thus made more commission errors. The results of this study again provide support to the notion that higher tendencies to make attentional errors, such as omissions and commissions in children with high-SCT symptoms may be due to their hypersensitivity toward salient threat-related information in the environment.

Barkley (2013) found that children with SCT had a greater association with lower parent education, lower income, and a greater likelihood of having a parent out of work due to disability than children with Attention Deficit Hyperactivity Disorder (ADHD). No previous research has been conducted to explain suck linkage. This study may provide a possible explanation of this linkage. The results of the present study suggested that freezing reaction (an increase of parasympathetic activities during warning signal condition) was related to the attentional errors (commission errors) in children with high-SCT symptoms. Previous studies have shown that individuals with adverse previous experiences, such as trauma were found to have a stronger freezing reaction toward stressful stimulus (Hagenaars et al., 2012; Niermann et al., 2015). It is possible that the social adversities associated with SCT may exacerbate the freezing response in these children, which causes more attentional problems and subsequently negatively impact their performance in real life activities.

So far, it is unclear why children with high-SCT symptoms have a higher tendency to have daydreaming in real life. The results of this study may provide a possible explanation and a direction for further research: their daydreaming tendencies may be due to their over-sensitivity toward threat-related information in the environment, which in turn causes more freezing-like behavior in them which is similar to the behavioral manifestation of daydreaming and mind-wandering. Such freezing-like behavior may affect their way to respond to the event in the real life. For example, they may divert attention toward the salient stress-related information but fail to attend to the inconspicuous but important goal-related information in the environment. As a result, they may fail to make appropriate responses to their real-life events. This is consistent with the results that individuals made more commission errors when they had stress-induced mind-wandering (Smallwood et al., 2009).

### Limitations of this study

One of the limitations of this study was the small sample size. Although it may undermine the significance of this study, the results of this study provided initial evidence that the attention difficulties exhibited by children with SCT were associated with their abnormal neurophysiological responses toward challenge/stressful stimulus. It may shift the focus of future research in SCT from purely cognitive domains such as attention and executive functions toward emotional state and regulation domains. By doing so, we will have a more comprehensive understanding of the nature of the SCT symptoms.

Another limitation of this study was that the results of this study were correlational in nature. It could not provide empirical evidence on the causal relationship between BIS sensitivity, anxiety, and attention difficulties in children with SCT. Future studies should compare the high-/low-SCT group difference in the neurophysiological state and regulation (theta wave, HRV, and electrodermal activity) in the attentional control task during high- vs. low-stress conditions. By manipulating the group status (high vs. low SCT) and the stress level (high vs. low stress), we will be able to provide direct evidence to support SCT as the cause of the abnormal physiological changes and behavioral responses during high-stress situations and to confirm the role of emotional regulation deficits in SCT symptomology.

### Significance of this study

The results of this study linked physiological dysfunction with behavioral difficulties in explaining the nature of SCT. These results not only can help us to understand the deficits of SCT at both physiological and behavioral levels, but also help us to understand the relationship between these two levels. With these results, we will be able to formulate a neuroscience model of SCT that can inform us on the clinical management of this disorder. This study identified hypersensitivity toward the stressful situations as the underlying mechanism explaining the attention problems in SCT. Therefore, it provides a scientific basis to support utilizing new treatments in SCT. For example, anxiolytic drugs may be a plausible medication treatment for SCT. In fact, previous research has shown that atomoxetine, which is an NE reuptake inhibitor and is frequently used as a treatment for depression and anxiety, could significantly improve the SCT symptoms in patients.

To conclude, this study has found that higher theta wave and higher SD1 nu in warning signal conditions were associated with CCPT Omission Errors and CCPT Commission Errors respectively. These results suggested that hypersensitivity toward stressful sensory stimuli is associated with attention difficulties in children with high-SCT symptoms. Therefore, SCT should not only be conceptualized as a condition resulting from cognitive deficits such as attention deficits. Instead, SCT should be conceptualized as a condition characterized by multiple deficits in different biological systems, such as the cognitive system, negative valence system, and arousal regulatory system. Without understanding such multiple facets of this condition, we will hardly be able to understand the nature of this condition and devise an effective treatment for it.

## Data availability statement

The data that support the findings of this study are not publicly available due to privacy issues and are available from the corresponding author upon reasonable request.

## Ethics statement

The studies involving human participants were reviewed and approved by the Hong Kong Polytechnic University and the ethics approval reference number is HSEARS20150724002-03. Written informed consent to participate in this study was provided by the participants’ legal guardian/next of kin.

## Author contributions

TY wrote the manuscript. CL reviewed the manuscript. TY, CL, and CC were responsible for the research design. TY and CL were responsible for the data collection. All authors contributed to the article and approved the submitted version.
